# Supplementary material for: Meta-analysis of the effectiveness and safety of Shenyankangfu tablets combined with losartan potassium in the treatment of chronic glomerulonephritis
Source: PLoS One. 2022 Oct 10;17(10):e0275735. doi: 10.1371/journal.pone.0275735 (PMC9550056; doi:10.1371/journal.pone.0275735)
Supplement: S1 File — (DOCX) [file pone.0275735.s003.docx]

Table S1 Comparison of 24-hour urine protein quantification in both groups before and after treatment（$\bar{X}$ ± s ）

| Study (years) | 24-hour urine protein quantification | |
| --- | --- | --- |
|  | Study group | Control group |

| Chen SY 2021 | 1.48±0.25 | 2.35±0.35 |
| --- | --- | --- |
| Guo YM 2018 | 1.26±0.35 | 2.03±0.58 |
| Li HX 2017 | 0.66±0.37 | 0.93±0.5 |
| Li XD 2017 | 0.66±0.37 | 0.95±0.51 |
| Lv TH 2020 | 0.7±0.2 | 1±0.5 |
| Pan HX 2019 | 0.92±0.23 | 1.24±0.42 |
| Qiu H 2017 | 0.65±0.36 | 0.92±0.49 |
| Qiu J 2018 | 0.31±0.12 | 0.82±0.64 |
| Su Y 2019 | 1.62±1.23 | 3.02±1.47 |
| Wang XL 2018 | 0.81±0.12 | 1.16±0.19 |
| Yan H 2018 | 0.31±0.12 | 0.82±0.64 |
| Yu GA 2017 | 0.36±0.09 | 0.54±0.17 |
| Zhao D 2017 | 1.5±1 | 2.2±1 |
| Zheng BL 2014 | 0.66±0.37 | 0.93±0.5 |

Table S2 Comparison of Serum creatinine quantification in both groups before and after treatment（$\bar{X}$ ± s )

| Study (years) | Serum creatinine | |
| --- | --- | --- |
|  | Study group | Control group |

| Chen SY 2021 | 90.35±6.55 | 106.35±8.25 |
| --- | --- | --- |
| Li HX 2017 | 91.4±16.2 | 92.1±18.6 |
| Li XD 2017 | 75.4±10.31 | 73.25±11.82 |
| Pan HX 2019 | 68.11±14.27 | 75.01±18.56 |
| Qiu H 2017 | 92±18.5 | 91.3±16.1 |
| Qiu J 2018 | 63.2±2.8 | 62.8±3.1 |
| Su Y 2019 | 69.88±12.32 | 87.54±15.62 |
| Wang XL 2018 | 91.41±9.04 | 97.7±14.23 |
| Wu Y 2016 | 109±30 | 109±35 |
| Yan H 2018 | 63.2±2.80 | 62.8±3.1 |
| Zhao D 2017 | 91±8 | 106±9 |
| Zheng BL 2014 | 91.4±16.2 | 92.1±18.6 |

Table S3 Comparison of blood urea nitrogen quantification in both groups before and after treatment（$\bar{X}$ ± s )

| Study (years) | blood urea nitrogen | |
| --- | --- | --- |
|  | Study group | Control group |

| Chen SY 2021 | 5.85±0.75 | 7.1±0.6 |
| --- | --- | --- |
| Li HX2017 | 5.98±1.32 | 5.9±1.12 |
| Li XD 2017 | 6.4±0.89 | 5.94±1.01 |
| Qiu H 2017 | 5.89±1.11 | 5.97±1.31 |
| Qiu J 2018 | 5±1.3 | 6±1.2 |
| Su Y 2019 | 2.13±1.01 | 3.98±1.56 |
| Wu Y 2016 | 8±2.2 | 8.1±2.1 |
| Yan H 2018 | 63.2±2.8 | 62.8±3.1 |
| Zhao D 2017 | 6±1 | 7±1.1 |
| Zheng BL 2014 | 5.98±1.77 | 5.9±1.12 |

Table S4 Comparison of Urine NAG enzyme in both groups before and after treatment（$\bar{X}$ ± s )

| Study (years) | Urine NAG enzyme | |
| --- | --- | --- |
|  | Study group | Control group |
| Chen SY 2021 | 15.05±6.30 | 25.30±5.75 |
| Li HX 2017 | 15.2±10.2 | 23.6±10.4 |
| Qiu H 2017 | 15.1±10.1 | 23.5±10.3 |
| Zhao D 2017 | 15±8 | 24±9 |
| Zheng BL | 15.2±10.2 | 23.6±10.4 |

Table S5 Comparison of leukotactin-1 in both groups before and after treatment

| Study (years) | leukotactin-1 | |
| --- | --- | --- |
|  | Study group | Control group |

| Chen SY 2021 | 71.85±10.55 | 135.45±18.45 |
| --- | --- | --- |
| Guo YM 2018 | 72.02±10.48 | 135.25±20.95 |
| Su Y 2019 | 69.38±20.02 | 98.56±19.63 |
| Wang XL 2018 | 96.08±23.67 | 128.17±34.03 |
| Wu Y 2016 | 71±20 | 132±44 |
