# Supplementary material for: Meta-analysis of the effectiveness and safety of Shenyankangfu tablets combined with losartan potassium in the treatment of chronic glomerulonephritis
Source: PLoS One. 2022 Oct 10;17(10):e0275735. doi: 10.1371/journal.pone.0275735 (PMC9550056; doi:10.1371/journal.pone.0275735)
Supplement: S2 File — (DOCX) [file pone.0275735.s004.docx]

**Efficacy and safety evaluation of Losartan potassium tablets and Shenyan Kangfu tablets in the treatment of proteinuria in chronic glomerulonephritis**

Chen SY^1^, Yang LY^1^, Huang Q^1^

1. Department of Pharmacy, Fourth people's hospital of Zhanjiang city, Zhanjiang, GuangDong province, China.

**Abstract：**

**Objective:** To explore the efficacy and safety evaluation of the combined use of Losartan Potassium Tablets and Shenyan Kangfu Tablets in the treatment of chronic

glomerulonephritis proteinuria. **Methods:** 80 patients with chronic glomerulonephritis proteinuria were selected and the random drawing method was selected for grouping treatment. There were 40 cases in both the control group and the experimental group, The trials were completed between June 2018 and June 2019. The control group received Losartan Potassium Tablets, while the experimental group received Losartan Potassium Tablets + Shenyan Kangfu Tablets. The clinical treatment effect, related indicators, cytokine levels before and after treatment, and adverse reactions were compared between the two groups. **Results:** The total effective rate of clinical treatment in the experimental group was higher than that in the control group (P<0.05). The 24h urinary protein content, urinary NAG enzyme, blood urea nitrogen and serum creatinine of the experimental group after treatment were lower than those of the control group (P<0.05). The TNF-α, TGF-β and LKN-1 after treatment in the experimental group were lower than those in the control group (P<0.05). the total incidence of adverse reactions in the experimental group was lower than that in the control group (P<0.05). **Conclusion:** The treatment of chronic glomerulonephritis proteinuria with Losartan Potassium Tablets and Shenyan Kangfu Tablets can improve clinical symptoms, with high safety and overall curative effect.

**Efficacy of Shenyankangfu tablets combined with Losartan potassium in the treatment of chronic glomerulonephritis and its effect on serum cytokine levels and immune function**

Guo YM^1^

1. Department of Pharmacy, Henan Province Hospital of TCM, Zhengzhou, HeNan province, China.

**Abstract：**

**Objective**: To investigate the efficacy of Shenyan Kangfu Tablet combined with losartan potassium in the treatment of chronic glomerulonephritis and its effect on serum cytokine levels and immune function. **Methods:** A total of 126 patients with chronic glomerulonephritis were selected and randomly divided into a study group and a control group, 63 cases in each. The study group was given Shenyan Kangfu Tablets combined with losartan potassium treatment, and the control group was given losartan potassium treatment alone. The 24h urine protein levels, serum cytokine levels and immune function were compared between the two groups before and after treatment. **Results:** The urinary protein levels, TNF-α, TGF-β, LKN-1, CD4+, CD8+, CD4+/CD8+ in the study group were significantly improved 24 hours after treatment (P<0.05), and the degree of improvement was significantly better than that in the control group(P<0.05). **Conclusion:** Shenyan Kangfu Tablet combined with losartan potassium is effective in the treatment of chronic glomerulonephritis. It can effectively reduce proteinuria, improve the levels of cytokines TNF-α, TGF-β, LKN-1. At the same time, it can regulate T cell subsets and improve immunity, which is worthy of clinical application.

**Observation on efficacy of Shenyan Kangfu tablets combined with losartan potassium in the treatment of chronic glomerulonephritis**

Li HX^1^, Sun Y^1^, Zhang SQ^1^, Ji ZH^1^, Shu Z^1^, Zhao YH^1^

1. Department of Nephrology, Shenyang Medical College Affiliated Central Hospital, Shenyang, LiaoNing Province, China.

**Abstract:**

**Objective:** To investigate the efficacy of Shenyan Kangfu Tablets combined with losartan potassium in the treatment of chronic glomerulonephritis. **Methods:** A total of 240 chronic glomerulonephritis patients were randomly divided into control group and observation group, with 120 cases in each group. The control group was treated with losartan potassium, and the observation group was also treated with Shenyan Kangfu tablets. Comparison were made on treatment effect, β-N-acetyl glucosaminidase (NAG), urinary protein, urea nitrogen, serum creatinine, and occurrence of adverse reactions between two groups. **Results:** The observation group had obviously higher total treatment effective rate as 95.0% than 78.3% in the control group, and the difference was statistically significant (P<0.05). After treatment, the observation group had better NAG as (15.2±10.2) U/L, urine protein as (0.66±0.37)g than (23.6±10.4) U/L and (0.93±0.50) g in the control group, and the difference was statistically significant (P<0.05). Both groups had no statistically significant difference in urea nitrogen and serum creatinine levels (P>0.05). The observation group had lower incidence of adverse reactions as 8.3% than 17.5% in the control group, and the difference was statistically significant (P<0.05). **Conclusion:** Application of Shenyan Kangfu tablets and losartan potassium provides remarkable efficacy in treating chronic glomerulonephritis, and it can effectively control urine protein of patients. So it contains high security.

**Observation on Efficacy of Losartan Potassium Tablets Combined with Shenyankangfu Tablets in Treatment of Proteinuria of Chronic Nephritis Proteinuria**

Li MB^1^, Sun ZM^1^, Ren ZQ^1^, Dong PD^1^

1. Dept of Nephrology, Qianjiang Central Hospital, Qianjiang, HuBei Province, China

**Abstract:**

**Objective:** To probe in to the efficacy of losartan potassium tablets combined with Shenyankangfu tablets in treatment of proteinuria of chronic nephritis. **Methods:** 68 patients with proteinuria of chronic nephritis admitted in to Qianjiang Central Hospital from May 2014 to Jan 2016 were selected to be divided in to observation group and control group via the random drawing methods, with 34 cases in each. The control group were treated with losartan potassium tablets, while the observation group were given losartan potassium tablets combined with Shenyankangfu Tablets. The efficacy of two groups were observed. **Results:** The total effective rate of observation group was 94.12%, significantly higher than that of control group (79.41%), with significant difference (P<0.05). The incidence of adverse drug reactions of observation group was 8.82%, significantly higher than that of control group (17.65%), with statistically significant difference (P<0.05). **Conclusion:** The efficacy of losartan potassium tablets combined with Shenyankangfu Tablets in treatment of proteinuria of chronic nephritis is remarkable with few adverse drug reactions and high safety.

**Observation of Therapeutic Effect of Chronic Nephritis Treated with Shenyankangfu Tablets and Losartan Potassium**

Li XD^1^

1. Urology Department, People's hospital of Yixing city. Yixing, JiangSu Province, China.

**Abstract:**

**Objective:** To observe the efficacy of Shenyan Kangfu Tablets combined with losartan potassium in the treatment of chronic nephritis. **Method:** The clinical data of 68 patients with chronic nephritis were analyzed and randomly divided into control group and observation group. The patients in the control group were treated with losartan potassium, and the patients in the observation group were treated with Shenyankang tablet on the basis of the treatment in the control group, and the clinical therapeutic effects of the two groups were observed and compared. **Results:** The total effective rate of treatment in the observation group was significantly higher than that in the control group.The clinical symptoms (fatigue, edema, lumbar muscle soreness, abdominal pain) score, 24-hour urine protein, interleukin-6 (IL-6), and interleukin-18 (IL-18) in the observation group were significantly lower than those in the control group. The difference was statistically significant (P < 0.05). There was no significant difference in serum urea nitrogen and serum creatinine levels between the observation group and the control group (P>0.05). **Conclusion:** Shenyan Kangfu Tablet combined with losartan potassium is effective in the treatment of chronic nephritis. It can effectively reduce urinary protein and has immunomodulatory effects. This scheme is worthy of clinical promotion and application.

**Study on the effective rate of the treatment of chronic nephritis with Shenyankangfu tablets combined with Losartan potassium.**

Lv TH^1^

1. Urology Department, Hospital of China National Heavy Duty Truck Group Company, Jinan, ShanDong Province, China.

**Abstract:**

**Objective:** This study analyzed the efficacy of Shenyankang tablet combined with losartan potassium in patients with chronic nephritis. **Method:** A total of 88 patients with chronic nephritis received in this hospital from September 2017 to September 2019 were randomly selected as the evaluation objects. The control group was treated with losartan potassium. The observation group was treated with Shenyan Kangfu Tablets combined with Losartan potassium, and the final clinical efficacy of the two different treatment regimens was analyzed in detail. **Results:** After treatment, the comparison between the two groups showed that the total effective rate of patients in the observation group was significantly improved, and the indexes of interleukin-6, interleukin-8 and 24 h urine protein were significantly improved, and the adverse reactions caused by the medication were also less. The difference was statistically significant (P<0.05). **Conclusion:** The application of Shenyan Kangfu Tablets combined with losartan potassium in patients with chronic nephritis can improve the quality of life of the patients.

**Clinical efficacy analysis of losartan potassium tablets combined with shenyan kangfu tablets in the treatment of chronic nephritis syndrome proteinuria**

Pan HX^1^, Fang MR^1^, Tong HQ^1^, Li T^1^

1. Urology Department, Leshan People's Hospital, Leshan, SiChuan Province, China

**Abstract:**

**Objective:** To analyze the clinical efficacy of losartan potassium tablets combined with shenyan kangfu tablets in the treatment of chronic nephritis syndrome proteinuria. **Methods:** 100 patients with chronic nephritis syndrome with proteinuria who were treated in our hospital from January2018to January2019were included in the study. Subjects were randomly divided into control group and experimental group, with 50 patients in each group. Patients in the control group were treated with losartan potassium tablets,and patients in the experimental group were treated with losartan potassium tablets combined with nephritis rehabilitation tablets. The improvement of proteinuria related indicators,effective treatment and adverse reactions in the two groups were observed. **Results:** The improvement of proteinuria related indexes in the experimental group and the effectiveness of treatment were better than those in the control group (P <0.05). The incidence of adverse reactions in the two groups was not statistically significant (P >0.05). **Conclusion:** losartan potassium tablets combined with shenyan kangfu tablets for the treatment of chronic nephritis syndrome proteinuria has a good clinical effect,is conducive to reduce the amount of urine protein, creatinine content, so as to improve the effective rate of treatment,is conducive to improve the degree of satisfaction of patients,worthy of clinical promotion and practice.

**Observation of Therapeutic Effect of Shenyankangfu Tablet Combined with Losartan Potassium on Chronic Nephritis. Prevention and Treatment of Cardiovascular Disease**

Qiu H^1^

1. Urology Department, The first Hospital of Quanzhou city, Quanzhou, FuJian Province, China.

**Abstract:**

**Objective:** To observe the clinical efficacy of Shenyan Kangfu Tablets combined with losartan potassium in the treatment of chronic nephritis. **Methods:** 120 patients with chronic nephritis who came to our hospital for treatment from January 2016 to March 2017 were selected by convenience sampling method, and divided into two groups by random number table method, with 60 patients in each group. Both groups of patients were given oral losartan potassium for treatment, and patients in the observation group were given Shenyankangbo tablet on this basis. **Results:** The clinical treatment effect of the observation group was significantly better than that of the control group, and the difference was statistically significant (P<0.05). The urine protein, NAG enzyme, serum creatinine and blood urea nitrogen levels of the two groups of patients before treatment were similar, and the difference was not statistically significant (P>0.05). the urine protein and NAc enzyme levels of the observation group were significantly improved after treatment , and its improvement was significantly better than that of the control group, and the difference was statistically significant (P<0.05). The incidences of adverse reactions such as dizziness and tinnitus in the observation group were significantly lower than those in the control group, with statistical significance (P<0.05). **Conclusion:** Shenyan Kangfu Tablets combined with losartan potassium in the treatment of chronic nephritis can significantly control the urinary protein of patients, with significant curative effect, fewer adverse reactions and high clinical safety.

**Observation of the curative effect of Shenyankangfu tablets combined with Losartan** **potassium tablets in the treatment of chronic glomerulonephritis and proteinuria**

Qiu J^1^, Li H^1^, Xing YX^1^

1. Urology Department, Shanxi Hospital of Integrated Traditional and Western Medicine,Taiyuan, ShanXi Province, China.

**Abstract:**

**Objective:** To probe in to the efficacy of losartan potassium tablets combined with Shenyankangfu tablets in treatment of proteinuria of chronic nephritis and proteinuria. **Methods:** 60 patients with proteinuria of chronic nephritis admitted in to Shanxi Hospital of Integrated Traditional and Western Medicine from Jan 2017 to July 2017 were selected to be divided in to observation group and control group via the random drawing methods, with 30 cases in each. The control group were treated with losartan potassium tablets, while the observation group were given losartan potassium tablets combined with Shenyankangfu Tablets. The efficacy of two groups were observed. **Results:** The total effective rate of observation group was 90%, significantly higher than that of control group (70%), with significant difference (P<0.05). After treatment, the 24h proteinuria, serum creatinine (Scr) and blood urea nitrogen (BUN) in the observation group were lower than those in the control group, and the difference was statistically significant (P<0.05). **Conclusion:** Shenyan Kangfu Tablets combined with Losartan Potassium Tablets has definite curative effect in the treatment of chronic nephritis with proteinuria, improves the clinical symptoms of patients, and is worthy of the majority of clinicians to use.

**Efficacy of Shenyankangfu tablets combined with Losartan potassium in the treatment of chronic glomerulonephritis and its effect on serum LKN-1, IL-1β and IL-1 levels**

Su Y^1^, Song K^2^

1. Nephrology rheumatology department, Angang Staff General Hospital, Anyang, HeNan Province, China.
2. Rheumatism Immunity Branch, Hospital of Traditional Chinese Medicine of Anyang City, Anyang, HeNan Province, China.

**Abstract:**

**Objective:** To explore the efficacy of Shenyan Kangfu Tablets combined with losartan potassium in the treatment of primary chronic glomerulonephritis and its effect on serum leukotactin-1 (LKN-1), interleukin-1β (IL-1β) and interleukin-1 (IL-1). **Methods:** According to the random number table method, 120 patients with chronic nephritis were divided into study group (Shenyan Kangfu Tablets + Losartan potassium, n = 60) and control group (losartan potassium, n = 60). The improvement of renal function indexes (serum creatinine (SCr), glomerular filtration rate (GFR), blood urea nitrogen (BUN), 24h urine protein) of the two groups of patients was observed, and serum LKN-1, IL-1β and IL-1 were detected. **Results:** After 3 months of treatment, the total effective rate of the study group was 83.33%, which was significantly higher than that of the control group, 75.00% (P < 0.05). The levels of serum LKN-1, IL-1β, IL-1 and some renal function indexes (BUN, SCr, 24h urinary protein) in the two groups were significantly lower than those before treatment (P < 0.01), and the study group was lower than the control group (P < 0. 05). GFR levels did not change significantly compared with those before treatment (P > 0. 05). **Conclusion:** Shenyan Kangfu Tablets combined with losartan potassium can effectively regulate the levels of serum LKN-1, IL-1β and IL-1 in patients with chronic nephritis, which is of great significance for promoting the recovery of renal function and improving the therapeutic effect.

**Treatment of Chronic Glomerulonephritis with Shenyankangfu Tablets Combined with Losartan Potassium**

Wang XL^1^, Xu K^2^, Lin SY^1^

1. Department of neurology, Xuwen County People's Hospital, Zhanjiang, GuangDong Province, China.
2. Department of urology, Xuwen County People's Hospital, Zhanjiang, GuangDong Province, China.

**Abstract:**

**Objective:** To investigate the efficacy of Shenyan Kangfu Tablets combined with losartan potassium in the treatment of chronic glomerulonephritis and the effect on serum leukotoxin-1 (LKN-1), tumor necrosis factor and interleukin-8 (IL-8). **Method:**

A total of 80 patients with chronic glomerulonephritis who were treated in our hospital from March 2015 to March 2017 were enrolled in the study and grouped according to the random data table method. Forty cases in the control group were treated with losartan potassium, and 40 cases in the observation group were combined with Shenyankangbo tablets, a course of 3 months. The renal function and inflammatory indexes of the patients before and after treatment were compared, and the difference in clinical efficacy between the two groups was compared. **Results:** After treatment, the 24h proteinuria, serum creatinine (Scr) and other renal function indexes in the observation group were lower than those in the control group, and the difference was statistically significant (P<0.05). The levels of LKN-1, TNF-yi and IL-8 in the observation group were lower than those in the control group (P<0.05). The total effective rate was 80.00% (32/40) in the control group and 95.00% (38/40) in the observation group, which were higher than those in the control group (P<0.05).

**Conclusion:** Shenyan Kangfu Tablets combined with losartan potassium in the treatment of chronic glomerulonephritis can better improve renal function and reduce inflammatory response, which is worthy of promotion.

**The Effect of Nephritis Rehａbilitａtion Tａblets Ｃombined with Losartan Potａssium on Serum Leukotaxine 1，Tumor Necrosis Factor αａnd Interleukin－33 of Patients with Chronic Glomerulonephritis**

Wu Y^1^, He Y^1^

1. Department of nephrology, Chongqing Fifth People's Hospital, Chongqing City, China.

**Abstract:**

**Objective:** To explore the effect of nephritis rehabitation tablet combined with losartan potassium on the serum leukotaxine 1 (LKN-1), tumor necrosis factor α (TNF α) and interleukin-33 (IL-33) of patients with chronic glomerulonephritis. **Methods:** Chosen between Sep.2011 and Nov.2011, 100 cases of chronic glomerulonephritis in Chongqing Fifth People's Hospital were included in the study. According to the random number table method, they were divided in to a control group and an observation group, 50 cases each. Both groups received conventional treatment (anti-infection and rest) and anti-coagulation treatment. The control group was added with losartan potassium, 50 mg per time, 1 time per day; the observation group on the basis of control group's regimen was added with nephritis rehabitation tablet treatment, 200 mg per time, 3 times per day. The curative effect, serum LKN-1, TNF α and IL-33 level, proteinuria and renal function improvement of the two groups were compared. **Results:** The total effective rate of the observation group was significantly higher than the control group (92% vs 68.0%). The serum LKN-1, TNF α and IL-33 level of the observation group were significantly lower than the control group. The proteinuria of the observation group was significantly lower than the control group. Renal function of the two had no statistically significant difference after treatment. **Conclusion:** The efficacy of nephritis rehabitation tablet combined with losartan potassium for chronic glomerulonephritis is good, which can effectively reduce serum LKN-1, TNF α and IL-33 level, thus is worthy of promotion in clinical.

**Clinical effect of Losartan Potassium and Shenyankangfu Tablets in the treatment of patients with chronic nephritis**

Xu ZY^1^

1. Department of neurology, Shehong County People's Hospital, Suining, SiChuan Province, China.

**Abstract:**

**Objective:** To study the clinical effect of losartan potassium and Shenyankangfu tablet in the treatment of patients with chronic nephritis. **Methods:** Chosen between July.2013 and August.2015, 100 cases of chronic glomerulonephritis in Shehong County People's Hospital were included in the study. All patients had normal renal function. They were divided in to a control group and an observation group. The control group was added with losartan potassium. the observation group on the basis of control group's regimen was added with Shenyankangfu tablet. The curative effect of the two groups were compared. **Results:** The total effective rate in the observation group was 96%, which was significantly higher than 80% in the control group; there was no statistically significant difference in the incidence of adverse reactions between the two groups. **Conclusion:** The clinical effect of losartan potassium and Shenyankangbo tablet in the treatment of patients with chronic nephritis is significant, and it is worthy of widespread promotion.

**Observation of the curative effect of Shenyankangfu tablets combined with Losartan potassium tablets in the treatment of chronic glomerulonephritis and proteinuria**

Yan H^1^

1. Blood purification room, Jiaocheng County People's Hospital, Lvliang, Shanxi Province, China.

**Abstract:**

**Objective:** To probe in to the efficacy of losartan potassium tablets combined with Shenyankangfu tablets in treatment of proteinuria of chronic nephritis and proteinuria. **Methods:** 70 patients with proteinuria of chronic nephritis admitted in to Shanxi Hospital of Integrated Traditional and Western Medicine from Jan 2017 to July 2017 were selected to be divided in to observation group and control group via the random drawing methods, with 35 cases in each. The control group were treated with losartan potassium tablets, while the observation group were given losartan potassium tablets combined with Shenyankangfu Tablets. The efficacy of two groups were observed. **Results:** The total effective rate of observation group was 92%, significantly higher than that of control group (73%), with significant difference (P<0.05). After treatment, the 24h proteinuria, serum creatinine (Scr) and blood urea nitrogen (BUN) in the observation group were lower than those in the control group, and the difference was statistically significant (P<0.05). **Conclusion:** Shenyan Kangfu Tablets combined with Losartan Potassium Tablets has definite curative effect in the treatment of chronic nephritis with proteinuria, improves the clinical symptoms of patients, and is worthy of the majority of clinicians to use.

**Analysis of the curative effect and immune function improvement effect of Shenyankangfu tablets in the treatment of patients with chronic nephritis**

Yu GA^1^, Yu XH^1^, Lv YZ^1^

1. Urology Department, The Third People's Hospital of Jingdezhen City, Jingdezhen, Jiangxi Province, China.

**Abstract:**

**Objective:** To explore the clinical efficacy of Shenyan Kangfu Tablets combined therapy in the treatment of patients with chronic nephritis and the improvement effect of their immune function. **Methods:** A total of 90 patients with chronic nephritis were selected and included in this study. They were divided into a combination group and a control group according to the order of admission. Both groups were given conventional treatments such as diuresis, antihypertensive, and anti-infection. On this basis, only losartan potassium was used to treat the patients in the control group, and the patients in the combination group were additionally treated with Shenyan Kangfu Tablets. After 3 months of treatment, the 24-hour urine protein quantification and serum IL-6 and IL-8 levels were compared between the two groups. **Results:** After 3 months of treatment, the 24-hour urine protein quantification in the combined group was (0.36±0.09) g, the serum IL-6 level was (2.36±0.57) ng/L, and the IL-8 level was (1.67±0.46) ng /L, were significantly lower than the control group, and the difference between the two groups was statistically significant (P<0.05). During the treatment period, there were no serious adverse reactions in the two groups. Among them, 2 cases of mild hyperkalemia, 2 cases of dizziness, and 1 case of cough occurred in the combination group, the incidence rate was 10.87%, and the incidence rate in the control group was 11.36%. There was no significant difference in the incidence of reaction, and clinical medication was relatively safe. **Conclusion:** Shenyan Kangfu Tablets combined with losartan potassium has a more significant decrease in urine protein level than losartan potassium treatment alone, and it can also control and regulate its immune function. It has good clinical efficacy and is worthy of active promotion.

**Efficacy of Losartan Potassium Tablets Combined with Shenyankangfu Tablets in Treating Proteinuria in Chronic Glomerulonephritis**

Zhao D^1^

1. Urology Department, Hospital of Traditional Chinese Medicine of Xinmin City, Shenyang, Liaoning Province, China.

**Abstract:**

**Objective:** To investigate the clinical efficacy of losartan potassium tablets combined with Shenyankang tablet in the treatment of patients with chronic glomerulonephritis proteinuria. **Methods:** 98 patients with chronic glomerulonephritis proteinuria who were admitted to Xinmin Hospital of Traditional Chinese Medicine from January to December 2016 were selected as the research subjects. According to the time of admission, the patients were divided into the control group (49 cases) and the observation group (49 cases) by the random control average method. The control group was treated with Losartan Potassium Tablets. The observation group was treated with Losartan Potassium Tablets combined with Shenyankang Tablets, and the therapeutic effects of the two groups were compared. **Results:** The total effective rate of treatment in the observation group was significantly higher than that in the control group, and the difference was statistically significant (P<0.05). The 24-hour urine protein quantification, systolic blood pressure, serum creatinine, blood urea nitrogen and urine N-acetyl-β-D-glucosidase (NAG) levels in the observation group were significantly lower than those in the control group, and the differences were statistically significant ( P < 0.05). **Conclusion:** Shenyan Kangfu Tablets combined with Losartan Potassium Tablets has a significant effect in the treatment of chronic glomerulonephritis proteinuria, and can effectively improve the levels of urinary protein, blood pressure, creatinine, blood urea nitrogen and urinary NAG in patients.

**Observation of Therapeutic Effect of Shenyankangfu Tablet Combined with Losartan Potassium on Chronic Nephritis**

Zheng BL^1^, Li T^1^, Yu JW^1^, Zhang XJ^1^, Guo QH^1^, Yang TG^1^, Liu BL^1^, Yang J^1^

1. Urology Department, Hospital of Traditional Chinese Medicine of Foshan City, Foshan, Guangdong Province, China.

**Abstract:**

**Objective:** To observe the clinical effect of Shenyan Kangfu Tablets combined with losartan potassium in the treatment of proteinuria in chronic nephritis. **Methods:** 82 patients with chronic nephritis with normal renal function were randomly divided into losartan potassium group and Shenyan Kangfu tablet combined with losartan potassium group. 24h urine protein quantification, urine N-acetyl-β-D glucosaminidase (NAG enzyme), serum creatinine and cytokines interleukin-6 (IL-6) and interleukin-18 were detected before and after treatment in the two groups respectively. **Results:** After 3 months of treatment, the total effective rate of the combined group was 80.5%, and that of the losartan potassium group was 61.0%. Comparing the two groups, the combined group was more effective than the losartan potassium group (P<0.05). The 24-hour urine protein and urine NAGase in the combined group were significantly lower than those in the losartan potassium group ( P < 0.05 ), and there was no significant difference in serum creatinine and blood urea nitrogen levels between the two groups (P>0.05). In terms of cytokines, the improvement of IL-6 and IL-18 in the combined group was significantly better than that in the losartan potassium group (P<0.05). **Conclusion:** Shenyan Kangfu Tablets combined with losartan potassium can more effectively reduce proteinuria in patients with chronic nephritis than losartan potassium alone, and has an immunoregulatory effect.
