# Supplementary material for: Meta-analysis of the effectiveness and safety of Shenyankangfu tablets combined with losartan potassium in the treatment of chronic glomerulonephritis
Source: PLoS One. 2022 Oct 10;17(10):e0275735. doi: 10.1371/journal.pone.0275735 (PMC9550056; doi:10.1371/journal.pone.0275735)
Supplement: Attachment — Submitted filename: Response to Reviewers.docx [file pone.0275735.s005.docx]

The revised manuscript is improved. However, few points remain to be addressed,

namely, i) need to elaborate more on RAAS blockers on lowering intraglomerular hypertension, direct podocyte effects by blocking ATII receptors;

Response: Thanks for your professional suggestion. Losartan potassium, which is an angiotensin II receptor antagonist can selectively compete for angiotensin receptor 2 binding that dilates renal arteries and blood vessels throughout the body, reducing proteinuria and blood pressure.

ii) need to provide more in depth details about the mechanism of action of Shenyankangfu tablets;

Response: Thanks for your professional suggestion. We have provided more in depth details about the mechanism of action of Shenyankangfu tablets. the Shenyankangfu Tablet (SYKFT) treatment of GN primarily involved infection, inflammation, or immunity processes. Considering that GN was primarily correlated with infection or autoimmune abnormalities, SYKFT may exert therapeutic effects by regulating pathways involved in immunity, inflammation, and oxidative stress [1]. In addition, treatment of CKD relies on effective control of proteinuria and protection of kidney function. In previous research, SYKFT was shown to provide a greater advantage in improving CKD clinical symptoms, controlling proteinuria, protecting kidney function, and reducing Western medicine side effects [2].

1. Jin M, Ren W, Zhang W, et al. Exploring the Underlying Mechanism of Shenyankangfu Tablet in the Treatment of Glomerulonephritis Through Network Pharmacology, Machine Learning, Molecular Docking, and Experimental Validation. Drug Des Devel Ther. 2021;15:4585-4601.
2. Lu H, Tang S, Su B. Clinical observation on treating 50 cases of chronic nephritis by Shenyan Kangfu tablets plus valsartan. Clin J Chin Med. 2012;4:79–80. doi: 10.4236/cm.2013.43012.

iii) need minor revision regarding grammatical errors.

Response: We sincerely appreciate the valuable comments. We found a professional language editing company to revise grammatical errors of the manuscript, and the proof is as follows:
